# Logical fallacies and invasion biology

**DOI:** 10.1007/s10539-018-9644-0

**Published:** 2018-09-06

**Authors:** Radu Cornel Guiaşu, Christopher W. Tindale

**Affiliations:** 1 Biology Program and Environmental and Health Studies Program, Department of Multidisciplinary Studies, Glendon College, York University, 2275 Bayview Avenue, Toronto, ON M4N 3M6 Canada; 2 Department of Philosophy, University of Windsor, 401 Sunset Ave., Windsor, ON N9B 3P4 Canada

**Keywords:** Invasive species, Logical fallacies, Native range, Invasion biology, “The straw man” fallacy, Non-native species

## Abstract

Leading invasion biologists sometimes dismiss critics and criticisms of their field by invoking “the straw man” fallacy. Critics of invasion biology are also labelled as a small group of “naysayers” or “contrarians”, who are sometimes engaging in “science denialism”. Such unfortunate labels can be seen as a way to possibly suppress legitimate debates and dismiss or minimize reasonable concerns about some aspects of invasion biology, including the uncertainties about the geographic origins and complex environmental impacts of species, and the control programs against species perceived as “invasive”. In assessing the quality of the debate in this area, we examine the validity of the use of various strategies, including the “straw man” concept, and explore a range of potential logical fallacies present in some recent prominent discussions about invasion biology and so-called “invasive” species. The goal is to add some clarity to the concepts involved, point out some problematic issues, and improve the quality of the debates as the discussions move forward.

## Introduction

Invasion biology is a relatively young and rapidly growing field. Although the origins of invasion biology are often traced back to the publication of the book *The ecology of invasions by animals and plants* by British ecologist Charles Elton in 1958, in reality the discipline itself did not really take off until the early 1990s, more than three decades later. The vast majority of invasion biology studies have been published within the last two and half decades, and the flagship journal *Biological Invasions* did not appear until 1999 (Ricciardi and MacIsaac 2008; Davis 2009; Simberloff 2011a). However, as this new discipline keeps expanding and becoming more influential, there have been growing criticisms of some aspects of invasion biology from biologists (for example: Slobodkin 2001; Davis et al. 2011; Thompson 2014; Guiaşu 2016; Schlaepfer 2018) and academics specializing in other areas (for instance: Rawles 2004; Sagoff 2005; Warren 2007), as well as science journalists (Burdick 2005; Marris 2011; Pearce 2015). The critics and criticisms made against invasion biology have generally been dismissed by prominent invasion biologists (Richardson and Ricciardi 2013). In particular, critics of invasion biology were accused of attacking a “straw man”, rather than the real field of invasion biology (Simberloff et al. 2011; Richardson and Ricciardi 2013), or coming up with a “red herring” to cast unjustified aspersions on the work of invasion biologists (Simberloff 2003; Richardson et al. 2008). More recently, Russell and Blackburn (2017) even stated that some critics of invasion biology were engaging in “science denialism”. This claim was vigorously disputed by a number of experts, including Crowley et al. (2017), Davis and Chew (2017), and Tassin et al. (2017), who suggested that the accusation made by Russell and Blackburn was unfounded and could be perceived as an attempt to shut down legitimate debates.

Such examples indicate that the tone of the debate is not always as professional as one would like to think or even expect. Indeed, as some of the exchanges to be discussed in this paper will indicate, the current state of debate on certain issues in this field might well be described as “unhealthy.” That is, the level of charges and dismissals is such that it will be very difficult for dialogues to move forward unless attempts are made to genuinely understand the positions being held and to clarify the terms involved. We strive in this article to offer such an attempt. At the same time, we identify and analyze a number of persistent problems which may affect our attitudes and approach towards species perceived (fairly or unfairly) as non-native, and impede genuine progress in our understanding of the multiple roles of these species in various ecosystems.

## Logical fallacies and debates about invasion biology

### “Straw men” and “red herring” fallacies

Some leading invasion biologists stated that critics of invasion biology attack so-called “straw men”, rather than genuine problems in this field (Simberloff et al. 2011; Richardson and Ricciardi 2013). However, in the rush to dismiss or diminish criticisms, invasion biologists may construct “straw men” of their own, and trivialize or downplay legitimate and thoughtful concerns, while ignoring specific examples and case studies that may not fit comfortably within the larger or predominant invasion biology narrative (Guiaşu 2016).

The “straw man” is one of a group of argument types traditionally identified as fallacies (Woods and Walton 1989; Tindale 2007). In this tradition, a “fallacy” is understood as an argument that appears to be valid but is not (Hamblin 1970). More recent treatments of fallacies have questioned the centrality of validity in any definition and included cases where arguments shift from one type of dialogue to another (Walton 1995), or any instance where the rules governing good argument are violated (van Eemeren and Grootendorst 2004). For our purposes, it is sufficient to recognize that any case where a conclusion is reached or a claim asserted in an inappropriate way can be considered fallacious.

Where there is a tendency in the debate to level such charges of fallacy against one’s opponents there is a particular need for caution. We might ask two things of this strategy: are the fallacies appropriately identified and charged, and do these charges help clarify positions in the debate? A discussion of several examples should illustrate these points.

Two popular fallacies in terms of the regularity with which they are employed and charged are Straw Man (or Person) noted above and Red Herring. Both are fallacies of diversion (Tindale 2007). The first diverts attention by misrepresentation; the second by an illegitimate shift of issue (without any misrepresentation). A “straw man” misrepresents another’s view by replacing that view with a caricature of it that is usually easier to attack. Then it takes the success of the attack on the caricature as a refutation or weakening of the actual position. This move in argumentation can be done deliberately (as we see so often in political exchanges), or unintentionally, as when someone fails to take the time to seriously consider what an opponent is saying and assumes something that is false (Talisse and Aikin 2006). By contrast, a “red herring” diverts attention away from an issue onto another for which the diverters have more information or which they find easier to handle, or to some unrelated aspect of the actual position. Both fallacies involve *irrelevance* insofar as what should be addressed is not addressed (Tindale 2007). As such, the presence of these fallacies in a debate is unhelpful because they do not provide for serious communication between those who disagree. Rather, they encourage antagonism and animosity. It is also important to observe that both of these fallacies are arguments. That is, there is a structure to each of them comprised of premises and conclusions. It is important to keep the difference between them clear. Consider the following:

In the conclusion to a paper defending attacks on introduced species, Daniel Simberloff (2003) writes:

This said, I must address what I believe is a red herring introduced by a philosopher (Sagoff 1999) and two ecologists (Slobodkin 2001; Rosenzweig 2001). This is the notion that current concern with introduced species is focussed on all introduced species and founded on the notion that introduced species are generically ‘bad’ and native species ‘good’. Although some extreme adherents of an aesthetic stance favoring native species doubtless hold such a view, invasion biologists do not, and the many recent government and international activities on introduced species explicitly recognize the enormous benefits of some introduced species. (Simberloff 2003, p. 189)

Now, perhaps Simberloff does believe there’s a red herring involved here.^1^ He may think that scholars like Sagoff have diverted the issue away from what is important and onto some other issue or, more likely, an irrelevant feature of the actual issue. But it seems more plausible that Simberloff believes Sagoff (and Slobodkin and Rosenzweig) have misrepresented the position held by Simberloff and those he identifies as “invasion biologists.” That is, the position Sagoff and others attack is not the position that most invasion biologists hold (in Simberloff’s view). We can identify the two positions:

1. (Attributed to Sagoff): the concern with introduced species (on the part of those who oppose them) involves *all* introduced species and promotes the dichotomy natives are good/introduced species are bad.
2. (Held by Simberloff and most “invasion biologists”): *some* introduced species are bad, but some are beneficial.

If Simberloff is correct about the opponents’ position, then those opponents are addressing a straw man. Thus, the disagreement is not being engaged as it should. But by identifying the error that is involved here we have a better sense of why that engagement is failing—not because of a diversion away from the issue, but because of a misrepresentation of what is at stake.

An example in a paper by Richardson and Ricciardi (2013) represents a similar case. At one point while drawing an analogy between false obituaries of humans and false reports of invasion science’s demise, they observe:

As one of us has argued previously, what many of the detractors write about is not invasion biology/ecology/science as understood and practised by almost all biogeographers, conservation biologists and ecologists (e.g. the definitions in Richardson et al. (2011) and the framework detailed in Blackburn et al. 2011), but rather a caricature or parody of the discipline (Richardson et al. 2008). (Richardson and Ricciardi 2013, p. 1462)

Here, Richardson and Ricciardi raise a concern about a straw man captured in the complaint about the caricature. Unlike Simberloff’s case, however, where features of the caricature were mentioned, here the claim is just made with a reference back to a brief paper by Richardson et al. (2008). So we do not actually see the argument that conveys the caricature with which Richardson and Ricciardi disagree. Again, in the interests of the debate itself readers should be presented with the argument in order to assess things for themselves. In its absence, the ability to “move forward and continue to build on the knowledge we have gained” (Richardson and Ricciardi 2013) is constrained.

What becomes apparent from these two simple examples, beyond the need to be clear about one’s terms, is that the nature of the debate itself changes according to who is presenting it. One important feature of the debate, then, is over the control of definitions and the descriptions of the disagreements themselves.

### The “Guilt by Association” fallacy

Guiaşu (2016) discussed the problems associated with negative perceptions of non-native species, in general, even in the absence of conclusive evidence against most of them. Such perceptions are often based on a few famous examples of invasive species, such as the brown tree snake in Guam or the zebra mussels in North America, which are known to cause certain problems in the ecosystems where they have been introduced. Discussions of new species introductions frequently begin with a reminder of the negative effects caused by these relatively few notorious invaders, and this creates the impression that any, or many, non-native species could cause devastating damage to the environment and native species, even though this is usually unlikely, and not proven, in most cases. This approach is obviously prejudicial, since it can generate hostile attitudes and lead to potential control programs against species which may be quite harmless, or may even, if given the opportunity, bring some positive contributions to their new surroundings. This is “Guilt by Association”, a type of Ad Hominem argument (as discussed by Tindale 2007), where a species can be attacked based on negative information gathered about a completely different and unrelated species, at a different location, and under different circumstances, simply because both species are considered to be non-native. The two species may have nothing in common, except for having a presumed non-native status.

In the logical literature, the Ad Hominem argument is literally “against the man”, and so it may be objected that it should not apply in cases where the associations are not between humans. But we can see that the principle involved is the same: there is an alleged association between parties (in this case species). One (or some) of those parties is (are) judged to be “guilty” in some way, essentially as an invader (or invaders) with damaging effects, and that guilt is then transferred to other associated species whose “guilt” was not determined or proven. We cannot dismiss the possibility that there are cases in which such a transfer would be reasonable, where an association does exist. But our point is that the association should not be simply assumed. There is a burden of proof on those who might reason this way to establish both the association and the “guilt” that is involved.

## Attitudes towards non-native species in invasion biology

### “Guilty until proven innocent”

The impact of non-native species is frequently complex and poorly understood and the “guilty until proven innocent” philosophy of invasion biology does not solve that problem. In fact, it extends the prejudicial nature of much of the debate by appearing to encourage what it actually precludes. As we saw in the previous section, prominent invasion biologists often defend their field by stating that they are not against all introduced species—just against the ones with a negative environmental impact (Simberloff 2003; Simberloff et al. 2011; Richardson and Ricciardi 2013). And yet there is in fact a shift of the burden of proof onto the so-called “invasive” species (or their supporters) that requires them to prove that they are not harmful.

The practical problem is that the nature of the environmental impact can often be just as difficult to classify in an objective and clear-cut way as the native or non-native status of various species (the difficulties associated with establishing native or non-native status will be analyzed in the “Additional problems in invasion biology” section of this article). Usually, species—native or introduced—have a variety of environmental impacts—some positive and some negative, depending on our points of view and which particular aspects and interactions we choose to focus on. There has been a general tendency in invasion biology to focus mainly on the negative impacts of species perceived as introduced. The positive impacts of non-native species, and the beneficial contributions these species can bring to ecosystems and native species, are frequently either minimized or ignored altogether in the invasion biology literature (Rodriguez 2006; Goodenough 2010; Guiaşu 2016; Schlaepfer 2018). Furthermore, if one sets out to “prove” at all costs that a species has some sort of a negative impact on the environment simply because the species exists—and therefore it must feed, occupy a certain space, use some resources, etc.—then one can “prove” that about any species anywhere, of course (Guiaşu 2016). Both native and non-native species can have complex ecological impacts and interspecific interactions, which are poorly understood in many cases due to insufficient research and mainly short-term rather than long-term studies (Willis and Birks 2006; Guiaşu 2016). Goodenough (2010) pointed out that “the ‘native good, alien bad’ maxim does not convey the complexity of invasion ecology: alien species do not axiomatically pose a threat to native biota.” Davis et al. (2011) also emphasized that both native and non-native species can have a variety of positive and negative effects on the environment, and, therefore, using the assigned native or non-native status of a species to determine (or pre-determine) its potential ecological impact is not very useful.

Aside from this practical concern there is a logical problem of equal import. Ruesink et al. (1995) deliver the clearest statement of this problem. For them, nonindigenous species represent a “major threat to the integrity of natural systems”. Their discussion does help to clarify how they would understand ‘threat’ and ‘major threat’, and in light of this discussion they express concern about a policy that they take to be “innocent until proven guilty,” where “taxa with no record of damage are generally considered acceptable imports.” But the difficulty of developing tests for such species and the high degree of uncertainty they see surrounding them push the authors to the other extreme: guilty until proven innocent. As they themselves admit, “even if we know the intimate details of species’ natural history and attributes and how these interact with new physical and biological surroundings, we cannot accurately predict the outcome of every introduction because the environment is complex.” This needs to be matched against their own “guilty until proven innocent” recommendation to fully see the import of what they are suggesting. They judge the most effective way to reduce harm is to assume that all nonindigenous species are threats. The authors thus recommend: “Keep nonindigenous species out of the United States *until a species is known to be safe*” (our italics). On the face of things, this seems like a clear requirement with a condition that must be met. But in practice, it is no condition at all because on the terms that the authors themselves have established it is a condition that can never be met. Recall: because the environment is complex, “we cannot accurately predict the outcome of every introduction” even when we have intimate knowledge of how an introduced species interacts with the environment. At some future time it is logically possible that a problem can emerge. Thus, a species can never be known to be safe with the degree of certainty those who propose the “guilty until proven innocent” policy require. And thus further, nonindigenous species cannot be proven innocent and so must always be deemed guilty.

There are various ways of presenting the fallacy at the heart of this policy. There is an element of “poisoning the well,” whereby an arguer presents a position in terms that predispose an audience against accepting it (Walton 1996). And there is the sense of a “false dilemma,” whereby an issue is presented in terms of a stark dichotomy without any appreciation of the middle ground between the extremes (Tomic 2013). However we understand this, we should appreciate the logical problem of presenting a condition that can never really be met. In drawing attention to this, we equally draw attention to the need for serious work to be done in the middle ground between the two extremes of assuming complete innocence and complete guilt.

Simberloff (2003) stated that the main reasons for concern about introduced species are that such species “can threaten the existence of native species and communities and that they can cause staggering damage, reflected in economic terms, to human endeavors.” Some introduced species may do that, but, then again, such problems may be caused just as readily by certain native species. For example, as Davis et al. (2011) pointed out, the native mountain pine beetle (*Dendroctonus ponderosae*) probably kills more trees in North America than any other insect species. Also, Venter et al. (2006) found that interactions among native species were more of a threat than introduced species to endangered species in Canada. In fact, introduced species were the least important threat among the six categories examined in that study. So, destructiveness and damage to the environment or the economy are not attributes associated solely with, or identifying features of, non-native species (Davis et al. 2011; Guiaşu 2016). Many introduced species are not guilty of causing any such environmental or economic problems.

In a survey of 422 invasion biologists—chosen because they acted as peer reviewers for the journal *Biological Invasions*—Young and Larson (2011) found that even among invasion biologists, the vast majority agreed that “There should be more scientific objectivity and less emotional xenophobia regarding invasive species.” An even greater majority of the invasion biologists surveyed agreed that scientists working in this field should “gather, interpret and communicate information” about invasive species “as accurately and objectively as possible.” These findings suggest that even many invasion biologists are aware that invasion biology is currently plagued by too much subjectivity, and presumably also too much unwarranted or insufficiently substantiated negativity regarding non-native species.

### The presence of bias

In a remarkably one-sided dismissal of the criticisms of what they called “invasion science”, Richardson and Ricciardi (2013) referred to the critics of their field as “a relatively small but vocal number of scientists and academics—naysayers in various guises” and declared that “many of the criticisms against invasion science simply do not withstand scrutiny.” It should be noted that this article was not a detailed review paper, but a rather brief editorial. This editorial was titled *Misleading criticisms of invasion science: a field guide*. Within the article, Table 1, which is labelled as “A field guide to misleading criticisms of invasion science”, summarizes six major criticisms of invasion biology and the so-called rebuttals to the criticisms, and provides a fairly small number of references, or sources, for each side of the argument. While, at first glance, this may appear to be an even-handed examination of these issues, it is quite clear that the authors of the editorial are far from neutral—in fact, they even refer to the articles they criticize as being part of “a cottage industry of criticisms”. The rebuttals are given in considerably more detail than the actual criticisms, and there is no way to decide, based on the limited information provided, whether the sources cited in support of the rebuttals provide comprehensive and convincing responses to each of the criticisms of the field. The impression given is that each of the criticisms listed in the table is without merit, and has been fully addressed and debunked in the relevant literature. However, that is not the case. In other words, the table about the supposedly “misleading criticisms” appears to be quite misleading itself. At least some of the already meagre sources cited for the rebuttals are very brief opinion pieces which do not offer any new data—for example, the Wilson et al. (2009) reference is a one page article published in the Letters Response category of the journal *Trends in Ecology and Evolution*, and the Simberloff et al. (2011) source is a very brief letter to the editor. Furthermore, plenty of additional studies and examples could have been selected, and a more detailed and nuanced description of the criticisms of invasion biology could have been included, of course. Another table could have been put together just as easily, showing studies and data which contradicted the rebuttals presented by Richardson and Ricciardi (2013).

In taking issue with the criticism that some control programs may be directed at non-native species that do not really cause any, or much, harm, Richardson and Ricciardi (2013) wrote:

In reality, managers are constrained by limited resources and seek to prioritize species that are likely to become problematic. However, this effort is hampered by several facts that are generally ignored by the naysayers: (1) the impacts of most invasions have not been studied, and so important effects may remain undetected, (2) invaders that are apparently innocuous in one region can be disruptive in other regions, (3) subtle impacts that may be unrecognizable without careful technical study can produce enormous ecosystem changes over time, and (4) many non-native species that currently appear innocuous may become damaging many years later – when it is no longer feasible to eradicate them. (Richardson and Ricciardi 2013, p. 1462)

The four items in this paragraph seem to fit within the earlier mentioned “guilty until proven innocent” philosophy. How do we decide which species “are likely to become problematic”, before any problems actually occur? What if we rush to judgement, and end up eradicating a species which would not have caused any harm, and may even have had a net positive impact, if we left it alone? Let us analyze the four so-called “facts” mentioned by Richardson and Ricciardi (2013), in the order in which they were listed above. (1) If the impacts of most invasions have not been studied, why should we necessarily assume that the effects of these introduced species will be important and negative? All that really means is that we don’t know anything about these species and their impacts. Consequently, unless we belong to that half of invasion biologists who believe invasive species have a negative impact by definition (Young and Larson 2011), we probably should not even call these species “invasive.” (2) Yes, theoretically, “invaders” may be harmless in one region and “disruptive” somewhere else. However, that clearly does not mean we have to consider non-native species automatically as problematic when they appear in a new area, particularly if these species are not known to have had a negative impact anywhere. It is also possible that even introduced species which have acquired a bad reputation elsewhere may cause no problems in the regions we are interested in. This is a two-way street. (3) “Subtle impacts that may be unrecognizable without careful technical study” may actually remain subtle, or minimal. “Careful technical study” may mean that we are trying a bit too hard, perhaps, to find some type of negative impact at all costs. This may not necessarily be the most efficient use of the “limited resources” mentioned by Richardson and Ricciardi above. Subtle impacts may, theoretically, produce larger impacts over long periods of time, but just how long would these projected periods have to be before we decide we must act now? If the impact of an introduced plant or animal will not be significant for hundreds or even thousands of years, it is obviously not of immediate concern. Who knows what current ecosystems will look like centuries or millennia from now? Many other factors will affect these ecosystems over such long periods of time. Furthermore, sometimes the impacts of invasive species become less important over time. So, again, this works both ways. In some cases, the impact of an invasive species may be most significant immediately after the introduction. After a few years, this impact may decline naturally, without our interference. This highlights the need for thorough and objective longer-term studies about the impact of non-native species, and suggests that we should be careful not to jump to (possibly premature) conclusions based only on very preliminary observations or short-term studies of such species (Guiasu 2016). And we should not start with the assumption that the “ecosystem changes over time” caused by non-native species have to be undesirable and harmful. (4) Yes, some non-native species may become “damaging” many years after their introduction, but, again, this is a theoretical concern that could be applied to any species. It is an a priori charge of “guilty”, or “potentially guilty one day”, without any supporting evidence. Changes in environmental conditions may cause a native species to become much more abundant and widespread than before as well. If we really care about managing our “limited resources” wisely, and if invasion biologists are really not against all introduced species (as Simberloff 2003; Simberloff et al. 2011; Richardson and Ricciardi 2013 stated), then it does not make sense to eradicate “non-native species that currently appear innocuous.” Do invasion and conservation biologists consider all non-native species to be problematic or not? Let us make up our mind, and let us all indicate clearly where we each stand on this. We cannot have it both ways. Quite simply, the “guilty until proven innocent” approach of some invasion biologists to non-native species, in general, seems incompatible with the assertion that these biologists are not against all such species.

Simberloff et al. (2011) stated that: “Pronouncing a newly introduced species as harmless can lead to bad decisions about its management.” So, does that mean that we can never declare a newly arrived species to be “harmless”, even in the absence of any evidence that would indicate otherwise? The assumption that a currently harmless species should be controlled anyway could also lead to potentially bad management decisions, resulting, for example, in the squandering of limited resources to fight against a species which should not be of concern and the damage to the environment that may be caused by such misguided control programs. Sometimes, actions taken against non-native species can have a variety of unintended consequences and may end up harming native species as well (Guiaşu 2016). For example, in New Zealand, poisons used to eliminate introduced mice also killed the North Island saddleback, a rare native forest bird (Davidson and Armstrong 2002).

In an article which attempted to find the middle-ground in some of the current debates about invasion biology, Shackelford et al. (2013) wrote: “Those who defend the removal of non-native species have been accused of xenophobia and those who are more ambivalent are charged with biological homogenization. Both sides have merit.”

In trying to analyze some current debates in invasion biology from a more neutral perspective, Shackelford et al. (2013) mentioned that the field “has shifted its rhetoric in recent years to reflect a focus on species with the greatest impact.” These authors also added:

However, how much of this shift reflects a change in attitude toward non-native species, rather than just limited resources and political appeal, is unclear. This point sits at the center of much recent controversy. Without resource and methodological constraints, many, if not most, conservationists would still probably prefer to rid systems entirely of non-natives regardless of impact. (Shackelford et al. 2013, p. 56)

In other words, if more resources were available, there is a risk that there could be more control and eradication programs against plant and animal species that may not have any proven significant negative impact, simply because these species are suspected of being non-native. Furthermore, the very concept of “impact” can be subjective in this field. If a species—for example, purple loosestrife—has been declared to be a problem, in general, it is very likely that control or eradication campaigns will be attempted against that species at many locations where its impact may not be negative overall. It is doubtful that conservationists or managers in charge of individual conservation areas are always conducting their own thorough independent research to determine whether the introduced species present within their jurisdictions are having mostly negative, or positive, impacts. Once a species is put on a list of undesirable “invasives”, it will probably stay there for a long time, even if convincing new exculpatory evidence may emerge. These lists are not always based on the best and most up-to-date science (Guiaşu 2016). And getting rid of non-native species entirely is clearly not a realistic goal, even assuming we could somehow identify all the non-native species in a given ecosystem, which would also be extremely unlikely.

### The value of informed dissent

In their editorial about the supposedly “misleading criticisms of invasion science”, Richardson and Ricciardi (2013) wrote: “To suggest that non-native species are not unequivocally a major concern for the conservation of biodiversity and ecosystem services is to ignore decades of peer-reviewed science.”

However, according to many ecologists (for example, Williamson 1996; Primack 2012), only a small minority of non-native species become invasive and cause problems, so why are we not allowed to think, or suggest, that many non-native species are not a concern for the environment or human interests? Moreover, researchers should not be accused of being ignorant of the relevant literature, willfully or otherwise, if they entertain such thoughts or make such suggestions and back them up with proper references and data. The word “unequivocally” seems rather misplaced in there as well. Again, the very definition of the term “non-native” is often unclear in this field, as will be discussed further in the next section, and even when we can actually determine that a species is indeed not native, the impact of that species is often ambiguous or poorly understood. In many cases, such species may have some negative short-term effects as well as some positive ones, and the long-term impacts may be unknown. So, to suggest that anyone who does not consider non-native species in general to be unequivocally bad or capable of causing major problems is ignoring “decades of peer-reviewed science” seems rather strange. As mentioned, the field of invasion biology is young, so we don’t have much more than about two and a half decades worth of peer-reviewed studies on display. And a thorough review of many of these studies, such as the one undertaken by Guiaşu (2016), can actually reveal that there is quite a bit of information that would contradict the assertion made by Richardson and Ricciardi (2013). Not all of these peer-reviewed articles point in the same direction or lead to the same conclusions. Perhaps it could be said that Richardson and Ricciardi (2013) are ignoring some of the peer-reviewed literature in their field when they are making such general statements. And some of the invasion biology papers, published in peer-reviewed journals, are mere opinion pieces. Such papers clearly add to the volume of the literature in this field, but they do not contribute any new evidence or data.

In their survey, Young and Larson (2011) found that 37% of invasion biologists agreed that “exotics are an unnatural, undesirable component of the biota and environment.” However, 34% of invasion biologists disagreed with that statement. This shows there is no unanimity, or even a strong consensus, in the field with respect to the presence and role of non-native species. Furthermore, in the same survey, 37% of invasion biologists agreed that “the term ‘invasive’ should not be used to connote negative environmental impact”, while 46% disagreed with that assertion. Young and Larson (2011) also found that there was almost equal support in the field for two different definitions of invasive species—one which stated that such species have negative environmental or socio-economic impacts and another one which did not. These findings contradict the assertion made by Russell and Blackburn (2017) that there is a “scientific consensus on the negative impacts of invasive alien species”. As discussed, species can often have a variety of impacts—some positive and some negative—and choosing which particular impacts to focus on may depend on our particular points of view or interests.

Therefore, it is perhaps unjustified and unfair to refer to critics of invasion biology as merely a few “naysayers”, who are ignoring the relevant scientific literature (Richardson and Ricciardi 2013), or members of a small “contrarian minority” (Simberloff 2011b), or individuals who are engaging in “science denialism” by rejecting “undisputed scientific facts” (Russell and Blackburn 2017). Invasion biologists themselves appear to be quite divided on some important issues and definitions in their own field.

Furthermore, not only is the notion of “scientific consensus” regarding the status and impact of non-native species far from clear, but the majority view at a particular point in time is not always invariably correct and should certainly not be above scrutiny.

For the most part, the criticisms of invasion science labelled as “misleading” by Richardson and Ricciardi (2013) seem quite legitimate and worthy of being carefully taken into account. According to some of these criticisms, as listed in Richardson and Ricciardi’s editorial, “impacts of non-native species on biodiversity and ecosystems are exaggerated”—presumably, the authors really meant to write “negative impacts”, rather than simply “impacts” in this case—and “positive (desirable) impacts of non-native species are understated”. However, the critics of invasion biology whose work we are familiar with are not saying that introduced species cannot have negative impacts or that these impacts are *always* exaggerated. Specific criticisms usually refer to particular species and examples. Nor can it be said that the negative impacts of non-native species are *never* exaggerated, or that the positive effects of these species are *never* ignored or understated in the invasion biology literature. Quite a few studies, including several cited in this article, would suggest otherwise. The overall scientific information on the impact of purple loosestrife in North American wetlands, for example, does not seem to justify the control programs and propaganda against this species throughout the continent (as shown in the comprehensive literature review by Guiaşu 2016). The impact of the rusty crayfish in Ontario is another relevant example. Do we know enough about it to start the anti-rusty crayfish campaigns? This species has been accused of having a negative impact on other crayfish species of the same genus. However, in a major study, Edwards et al. (2009) found that the widespread decline of all crayfish species sampled in many lakes in south-central Ontario during a couple of decades or so could not be attributed to the rusty crayfish. In fact, Guiaşu (2016) has shown that at least some of the anti-rusty crayfish information published in Ontario was based largely on some studies conducted in Wisconsin, and these studies were not always cited and interpreted in the proper context. Obviously, we should not use data about the possibly negative impact of a species in one location to demonize that species everywhere, especially since the impact of species can vary quite a bit from one location to another, as was demonstrated in the case of the rusty crayfish as well (Capelli 1982; Guiaşu 2016). Rarely is positive information about invasive species included in the brochures, information sessions, guided nature tours, or lectures associated with, or influenced by, this field. Nor do many invasion biologists or wildlife managers set out to find out just how much of a good contribution exotic species can bring to various ecosystems. What is the percentage of invasion biology studies which specifically focus on the positive attributes of non-native species? Without such detailed information, these criticisms of invasion biology cannot be labelled as “misleading.” In similar fashion, including major human diseases caused by viruses or bacteria on the lists of costs associated mainly with invasive animals and plants, as Pimentel et al. (2005) and Colautti et al. (2006) have done, could also be classified as potentially “misleading”. Non-native plants and animals generally do not have the same impacts as pathogens and dealing with illnesses which affect humans is the responsibility of medical professionals not invasion biologists. In addition, funding for health care is usually separate from funding for programs against invasive species (Guiasu 2016).

Richardson and Ricciardi (2013) tend to describe the chosen criticisms of invasion biology in very general terms. For example, one of the criticisms is described as “increased species introductions raise biodiversity (e.g. by adding to regional species pools; generating new taxa through hybridization) and therefore do not merit concern.” Well, introduced species can actually do that, and new varieties and species may eventually evolve as a result of interspecific hybridization, especially if we take a long-term evolutionary view, but that doesn’t mean the critics of invasion biology are always suggesting that invasive species are never of any concern. That is an overgeneralization, and, therefore, an exaggeration, or even a caricature, of the position taken by certain critics of the field. Just because some of us take a longer term and more generous view of non-native species and processes such as natural hybridization between native and exotic species, that does not mean we are oblivious to potential problems associated with the introduction of species. And it may be worth keeping in mind that non-native species deserve to be known and studied for more than the potential problems they may sometimes cause, and, as previously mentioned, native species are perfectly capable of generating environmental and economic troubles as well.

Richardson and Ricciardi (2013) cited only three studies (Brown and Sax 2004; Vermeij 2005; Thomas 2013) as sources for the “increased species introductions raise biodiversity and therefore do not merit concern” criticism. However, none of these three studies actually state that species introductions should not be of any concern, in general. In fact, Brown and Sax (2004) and Vermeij (2005) recognize that some non-native species can have negative effects, at least in the short term. These authors also clearly indicate that the introduction of such species should not be encouraged. And Thomas (2013) specifically mentioned that: “It is true that some invasive species damage ecosystems and can eradicate resident species.” He also added: “There are excellent arguments for conserving the wildlife we already have, but it is less clear why our default attitude to novel biodiversity is antagonism or ambivalence.” Furthermore, Thomas’s 2013 article is full of interesting specific examples which illustrate his point that biodiversity can indeed be increased as a result of species introductions. Overall, Brown and Sax (2004), Vermeij (2005), and Thomas (2013) encourage a more objective and open-minded approach to the study of non-native species and their impacts, based on evidence collected over the longer term, and not just the short term.

## Additional problems in invasion biology

### Cryptogenic species and the difficulties of separating native from non-native species

One of the fundamental, enduring, and much discussed problems in invasion biology is the rather arbitrary nature of key terms such as “native”, “non-native”, “invasive”, and “native range”.

Some authors consider introduced, or exotic, or non-native species to be those that have arrived in a particular area since the beginning of recorded human history, and native or indigenous species those that have either evolved in the region or moved there during prehistoric times (Myers and Bazely 2003). Webb (1985) attributed native status to those species which had been present in the U.K. and Ireland before the start of the Neolithic period, or have arrived since then without any human assistance.

The problem with these and other similar definitions is obvious. It is often difficult or impossible to determine exactly how or when a particular species reached a certain area. This is not just an academic discussion, since species labelled as “non-native” or “invasive” can be the targets of control or even eradication programs, while “native” species can be protected and promoted (Guiaşu 2008). A large chapter in a recent book (Guiasu 2016) was dedicated to the practical difficulties of separating “native” from “non-native” species in logically consistent ways in many cases, and it is clear that entire books could be written on this subject alone.

As one of many such examples, we can look at a recent debate about the status of some northern pike (*Esox lucius*) populations in Ireland. Until recently, this freshwater fish species was thought to have been introduced by people to Ireland during medieval times. Because of its presumed non-native status, and since the pike is a top predator, control programs against this fish were undertaken in Ireland to protect the native brown trout (*Salmo trutta*). However, a new genetic study has revealed that there are actually two distinct pike lineages in Ireland, and one of them may have reached Ireland without human help. The older and more widespread lineage may be native and unique to Ireland and may have originated about 4000 years ago, at the end of the last Ice Age in the region, while the other lineage may indeed have been brought to Ireland by people about 1000 years ago (Pedreschi et al. 2014). Ensing (2015) agreed that there are indeed two genetically distinct lineages of pike in Ireland, but hypothesized that both of these lineages could have been introduced by people to Ireland, several thousand years apart, and, therefore, both lineages would likely be non-native. Pedreschi and Mariani (2015) found Ensing’s argument unconvincing and maintained that the data analysis supported the native status of the older Irish pike populations which originated approximately 4000 years ago. The debate, which has potential management implications for the Irish pike populations, seemed to focus mostly on whether or not people had the technology and the knowledge required to possibly transport live freshwater fishes (such as pike) over long distances across the sea during the Neolithic. This would seem difficult to establish with much certainty, one way or another, and, furthermore, the timing of the retreat of the glaciers from the area now occupied by the Irish Sea is unclear, as is the exact nature of the post-glacial landscape of the region thousands of years ago (Pedreschi and Mariani 2015). While the scientific debate may have merit, one has to wonder why the outcome should make a difference to the management of pike in Ireland, and whether control programs can be justified in this case because of a perceived “non-native” status of this fish. Either way, pike have clearly lived in, and been an integral part of, Irish freshwater ecosystems for thousands of years, and it seems bizarre to persecute them now simply because of the possibility that their distant ancestors may have been brought to Ireland by people a very long time ago. We can legitimately ask how much time has to pass before we can finally accept a potentially introduced species as a “natural” part of local ecosystems? In many cases, we simply do not know if certain species were introduced at various locations by people, or arrived on their own, or both, because the evidence for any of these scenarios is either insufficient or non-existent (Guiaşu 2016).

Due to the difficulties of establishing native or non-native status for numerous species, Carlton (1996) introduced the concept of cryptogenic species. These are species whose geographic origins simply cannot be determined based on the available evidence. Therefore, such species, which, in Carlton’s words, can be “remarkably common” in many ecosystems, cannot be assigned either native or non-native status. Certain species may have both “introduced” and “native” status at the same time. For example, the coconut palm (*Cocos nucifera*) may have arrived in the Galápagos Islands on its own, since the fruits of this tree can be dispersed by oceanic currents over long distances, and with the help of people (Guiaşu 2016).

### The vague concepts of “native range” and “invasive”

It is also difficult to establish the exact boundaries of a “native range” for most species. As an example, we can look at the distribution of the rusty crayfish (*Orconectes rusticus*)—probably the most reviled crayfish species in North America (Guiaşu 2008, 2016). This crayfish is considered invasive in many parts of the continent, including the Canadian Province of Ontario, and is a target of control programs which aim to eliminate it from certain lakes and streams (Hein et al. 2006, 2007). However, the exact extent of the native range of the rusty crayfish is unclear. According to some leading experts, the native range of *O. rusticus* may well have included southern Ontario, as well as the American states of Michigan, Ohio, Indiana, Kentucky, and Tennessee (Hobbs 1989; Hobbs et al. 1989). Other authors give somewhat different native ranges for the rusty crayfish. For instance, Butler and Stein (1985) limit this native range to Indiana, Illinois, and western Ohio, while Mather and Stein (1993) substituted Kentucky for Illinois in the range given by Butler and Stein (1985).

The debates regarding the exact extent of the native range of the rusty crayfish continue. Recently, Dresser and Swanson (2013) described two different native ranges for this species. A more detailed analysis of the controversies associated with the identification of the boundaries of the native range for this and other species is given in the book by Guiaşu (2016). There are almost as many different versions of the “native” range of the rusty crayfish as there are studies mentioning this distribution.

The predominant current view appears to be that *O. rusticus* is an introduced species in Ontario, but a native one in parts of nearby Michigan and Ohio. Some biologists assume the rusty crayfish was introduced into Ontario by fishermen from the United States who used this species as bait. However, we really don’t know how this species first arrived in Canada. In fact, it is entirely possible that some of the range expansions of *O. rusticus* into, and within, Ontario were accomplished without human assistance. This resourceful crayfish species can survive quite well in a variety of aquatic habitats—from small ponds to fast streams—and is capable of dispersing itself very efficiently on its own (Guiaşu 2007, 2008). Furthermore, Peters et al. (2014) mentioned that the rusty crayfish was present in Lake Erie for more than a century. It is, of course, possible that the rusty crayfish may have been present in Lake Erie, and other locations, for much longer periods of time. Museum records indicate that scientific crayfish collection efforts in jurisdictions such as Ontario, for example, do not go back much longer than about one century (Guiaşu 2016). According to Peters et al. (2014), *O. rusticus* may be native to southwestern Lake Erie. If that is indeed the case, there would be nothing to stop the rusty crayfish from expanding its range on its own to various parts of the Great Lakes region of North America, including Ontario, through various interconnected bodies of water within this enormous and complex freshwater system. The distributions of species are clearly dynamic, and some rusty crayfish may well enter Ontario’s rivers and lakes on their own from neighbouring Michigan and Ohio right now, entirely undetected. There are no environmental barriers preventing them from doing so. Unfortunately for these crayfish, they have been labelled as “invasive” in Ontario already, based on very flimsy evidence, and so regardless of their future movements in this province, it is unlikely that invasion biologists will ever give these crayfish a chance to become a “natural” part of the local freshwater ecosystems (Guiaşu 2016).

Although Simberloff et al. (2011) and Richardson and Ricciardi (2013) seem to suggest that invasion biologists usually speak with one voice, this does not appear to be the case. Colautti and MacIsaac (2004) consider the term “invasive” to be confusing, and list five different definitions used in the invasion biology literature for this one term. In their previously discussed survey, Young and Larson (2011) found almost equal support for two alternative definitions of the term “invasive”. One of these definitions suggested that invasive species have a negative impact on the environment and the other one did not. But the question of terminology has a further problem beyond the prejudicial nature of some of the terms’ usage. Two terms of choice in the debate are noteworthy for what they connote. We have in mind here “invasive species” and “introduced species”. Both suggest agency.

With “invasive species,” it is the species themselves who are the agents. In the second case—“introduced species”—the agent is the introducer, usually a human. We need to think carefully about the adoption of either of these choices. Do we want the implication of agency here? One reason for not preferring it is that it invariably carries with it a sense of intentionality, which encourages us to think of the motives behind them. At least in the first case, this is inappropriate. But we do not need to go in the direction of agency when there is a far preferable, and thoroughly neutral, alternative. That is “non-native” species (bearing in mind the difficulties we have already discussed of establishing what are “non-native”). This label simply captures what they are (or may be) with no presumption of agency or intentionality.

A term like “invasive” should be seen as metaphorical. Metaphors are vehicles for meaning, transporting it across semantic divides. In this way, metaphors further serve to focus the mind in quite specific ways. Sometimes this focusing is deliberate; the user wants to invoke certain ideas and have the audience think accordingly. But given the ubiquity of so many metaphors in our language, they can be introduced and then propagated quite unintentionally. In the debate surrounding invasion science, we see examples of both.

As our discussion demonstrates, definitions definitely matter in this debate. Some of the scientists involved understand “invasive species” in a way that precludes the understanding of others. That is, some have argued (for example, Thomas 2013, 2015) that certain “invasive” species actually enhance biodiversity for example through hybridization and speciation processes, while at the same time maintaining vital ecosystem functions. Others (for instance, Simberloff 2003), however, define “invasive species” in a purely negative way such that no positive outcomes are possible. That is, “invasive species” are defined as those species that have a significant negative impact on the ecosystems in which they are introduced. This definition precludes any positive outcomes. This is another reason to prefer a more neutral term like “non-native.” In fact, definitions should be as neutral as possible unless they are describing something that has a value as part of its nature. In this debate, the question of value is what is at stake and needs to be argued. Defining things in a negative (or positive) way is one strategy for avoiding the argumentation altogether. But this is a serious violation of one’s obligations, and it will not advance the quality of discussion very far.

In fact, the term “invasive” is clearly prejudicial and the use of this term generates negative attitudes towards species carrying this label from the beginning. Larson (2011) discussed the inappropriate metaphors and militaristic rhetoric often used by invasion biologists and referred to this approach as the “advocacy by fear”.

The inconsistent use of key terms in invasion biology, as well as the lack of clear definitions for these terms, have been noted by a number of authors over the years (for example: Falk-Petersen et al. 2006; Guiaşu 2016). In a recent literature review, Pereyra (2016) mentioned that the use of value-laden terms, such as “invasive”, remains common in this field, and the definition for this term and the criteria used for assigning this status to particular species are often not found in many current invasion biology studies.

## Conclusions and implications

In his comprehensive book on invasion biology, Davis (2009) identified a number of problems associated with this field, including certain “issues of pluralism”, and noted that at times invasion biology “has not been as welcoming as it might have been of diverse perspectives.” In this section of his book, ecologist Mark Davis made a plea for accommodating more diverse perspectives in invasion biology as a way to strengthen and invigorate the field and make greater progress possible. However, as we have seen throughout the article, these types of issues persist in invasion biology almost a decade after the publication of Davis’ book, and even several legitimate and thoughtful criticisms of certain aspects of invasion biology tend to be dismissed or diminished by some leading invasion biologists. And accusing some critics of invasion biology of engaging in “science denialism”, as Russell and Blackburn (2017) have recently done, for example, is clearly not helpful for having a thorough and constructive debate in this field.

The kinds of problematic language strategies that we have examined have implications beyond those we have already identified. The practice of science is a multi-faceted and complex enterprise. But the discussion here does have consequences for how we understand one aspect of this practice. Science is touted as an objective discipline interested in discovery and descriptions. But those engaged in this discipline are human agents with all the foibles that attach themselves to such agents in whatever sphere they operate. For decades now science has been addressed as a rhetorical activity, with scholars keen to identify its rhetorical nature (Prelli, 1984; Gross 1990; Ceccarelli 2001). Gross (1990), for example, witnesses beneath the calm façade of the scientific paper a struggle to control meanings, attract followers, and establish authority. This, we have encountered in the debate over invasion science. What this study shows is how important it is to be aware of these “secondary” elements in the practice of science, to identify them and monitor their influence.

How scientists choose to use a term (and it is a choice), how they promote and control its meaning, will influence how they then see the world, which in turn will affect their research. Adopting the language of closure, as we have seen happen, so that debate is forestalled, narrows our vision of things so they appear as reflections of the language and in turn reinforce the fixed belief of the scientist. There is no merit in seeing things otherwise, because any alternatives have been dismissed by the language that describes them. Thus, when non-native species are judged guilty until proven otherwise, and at the same time the possibility of their being proved otherwise is discounted, then avenues of thought are closed. The openness that commonly characterizes effective scientific thinking is impeded.

It is not, then, just the deplorable quality of the debate that might concern us. There is an underlying threat to the ability of the field to fully weigh the merits of proposals advanced for consideration, whether in the evaluation of granting agencies or the peer-review process. Meanings affect the way things are valued, and disvalued concepts and processes are not considered worthy of serious attention.

Schlaepfer (2018) discussed the considerable difficulties and resistance encountered during the peer-review process when he submitted a manuscript in which the authors tried to present a more positive and balanced view of non-native species. The study, which was eventually published after overcoming many obstacles, and was criticized even after publication, carefully documented some of the positive contributions brought by non-native species. These notable contributions included providing habitat, shelter, and food for native species, ecosystem services (such as pollination of native plants and dispersal of native plant seeds by non-native animals, for example), and taxon substitution (when a non-native species takes over the ecological role of an extinct species, as was the case with some giant tortoise species on oceanic islands, for instance) (Schlaepfer et al. 2011).

Despite the undeniable positive contributions non-native species can bring (for instance, Guiaşu included an entire chapter on this topic in his 2016 book on non-native species), these positive contributions tend to be either minimized or ignored, for the most part, in the invasion biology literature, as discussed earlier in this article. This is likely often because at least some invasion biologists cannot bring themselves to say or write anything positive about non-native species, since this would go against the predominant narrative in a field which remains largely hostile towards, and deeply suspicious of, such species.

Kareiva and Marvier (2018) stated that: “In a field that frequently relies upon fear appeals to motivate action […] data that run counter to doom-and-gloom messages can be especially unwelcome.” Their comments referred to conservation science in general, but can also apply just as well to invasion biology, which is, after all, a branch of conservation biology. Silliman and Wear (2018) mentioned that:

Conservation science was from the start motivated by the mission of protecting biodiversity and is unusual among scientific disciplines in having a foundational set of normative principles […]. These principles morphed a bit over time in our collective conscience to be: native good, introduced bad; intact good, fragmented bad; and natural good, human-modified bad. (Silliman and Wear 2018, p. 182)

Invasion biologists and their supporters frequently defend actions taken against “invasive” species by citing the uncertainty about the potential effects such species may have on the environment. However, in our view, this uncertainty is not adequately addressed by the “guilty until proven innocent” approach that seems all too common in this field. The most logical reaction to uncertainty should be trying to gather more facts about the species in question and learn more about their impact and interactions, rather than jumping straight to control and even eradication programs. This is not only a more rational, open-minded, and compassionate approach, but also often a more practical one. The limited resources available for conservation should be spent wisely, not dogmatically, and sometimes, when new and more comprehensive data emerge and species are either exonerated or not conclusively proven to have negative effects, it may be useful to simply adjust our views accordingly, admit past mistakes, and let the actual evidence, instead of a general dislike of non-native species, guide our actions. If we don’t have enough good reasons to be at war with purple loosestrife, or other such so-called invasive species, then perhaps we shouldn’t be.

However, as Silliman and Wear (2018) recently stated, “the practice of conservation is, unfortunately, often not adaptive”, and this, according to these authors, results in “new knowledge and contrarian data being overlooked and not acted on.” Perhaps the time has come to rethink some of our assumptions in this field and have more flexible, case by case, data-based responses to various conservation-related issues.

Most non-native plants and animals are not expected to have catastrophic effects on the environment or the economy, so, in the vast majority of cases, we should have the time to properly assess the effects of these species and reach legitimate fact-based conclusions without having to rush to judgement. The burden of proof should be even greater for those who advocate potentially expensive, destructive, and sometimes gruesome control programs against a variety of animals and plants (Guiaşu 2016). Under these circumstances, it is not too much to ask that the evidence used to justify such programs should be clear, relevant, specific, and compelling.

Furthermore, the “guilty until proven innocent” mentality often prevalent in invasion biology may conveniently absolve researchers in this field from the essential responsibility of collecting adequate data to make the case against the species they are targeting. In addition, this a priori negative approach may prevent invasion biologists from properly taking into account the potentially positive effects non-native species may have on ecosystems (and the economy) and the mutually beneficial relationships among various native and non-native species. We do not have to treat the arrival of every non-native species as an unfortunate event. Keeping an open mind and expanding the focus of research on non-native species beyond just a relentless search for negative effects may yield interesting and surprising results, and ultimately lead to a healthier and more sustainable relationship with the natural world around us.

In trying to portray as positive an image of invasion biology as possible, Richardson and Ricciardi (2013) refer to it as “a valuable and thriving metadiscipline”, and mention that “one of the principal goals of the field—to predict which introduced species will become disruptive—is of increasing societal importance”. However, aside from the fact that we don’t know exactly what is meant by the term “disruptive”, which could be quite subjective, even the most dedicated proponents of invasion biology seem to agree that the ability to make such predictions is not one of the current strengths of the field. In fact, the abstract of a recent article (Kumschick et al. 2015) about the ecological impacts of alien species—a study with 19 authors, including Ricciardi and Richardson—begins with the following admission:

Despite intensive research during the past decade on the effects of alien species, invasion science still lacks the capacity to accurately predict the impacts of those species and, therefore, to provide timely advice to managers on where limited resources should be allocated. (Kumschick et al. 2015, p. 55)

We would be wise to heed the caution evident in such observations. The field is at a stage where no firm pronouncements should be made without clear and compelling evidence to support them. And, as we have seen, such evidence remains in short supply. In its place, we have found a number of rhetorical and logical moves that distort the tone of the debates involved and stand to impede the development of constructive dialogue in the field. It has been the modest goal of this paper to draw attention to this state of affairs in a way that will hopefully encourage balanced and respectful discourse as we move forward.

## Footnotes

1 In fact, at the time Sagoff, Slobodkin, and Rosenzweig wrote, many in the field did not make the distinction between good and bad species in this way. So, any attribution of a red herring would have been inappropriate. We are grateful to an anonymous reviewer for making this point.
